# A system for spatial hearing research

**DOI:** 10.1016/j.mex.2022.101727

**Published:** 2022-05-14

**Authors:** Arivudai Nambi Pitchai Muthu, Hasna Fathima, Vibha Kanagokar, Jayashree S. Bhat, Sathish Kumar

**Affiliations:** a Department of Audiology and Speech-Language Pathology, Kasturba Medical College, Mangalore, Manipal Academy of Higher Education, Manipal, Karnataka, India; b Consultant Audiologist and Speech-Language Pathologist, Kasturba Medical College Hospital, Ambedkar Circle, Mangalore, Karnataka, India; c Department of Audiology and Speech-Language Pathology, National Institute of Speech and Hearing, Trivandrum, Kerala-695017, India

**Keywords:** Binaural hearing, Sound localization, Spatial hearing lab

## Abstract

The spatial hearing experiments can be simulated using high-fidelity headphones. But these simulated experiments do not account for individual variations and are difficult to investigate when the listener is wearing hearing devices. Hence, the free-field systems are ideal for spatial hearing experiments. However, these systems are not readily available and must be customized based on experimental needs. This paper provides a brief overview of a spatial hearing research facility that is customized to perform experiments on individuals with normal hearing and hearing aid users.

• This setup enables the assessment of spatial acuity with 10⁰ precision in the horizontal plane.
• The laboratory's universal design enables modifications based on experimental needs with minimum effort.
• The signal processing and response acquisition systems are custom designed using MATLAB.

## Graphical abstract

**Figure:**
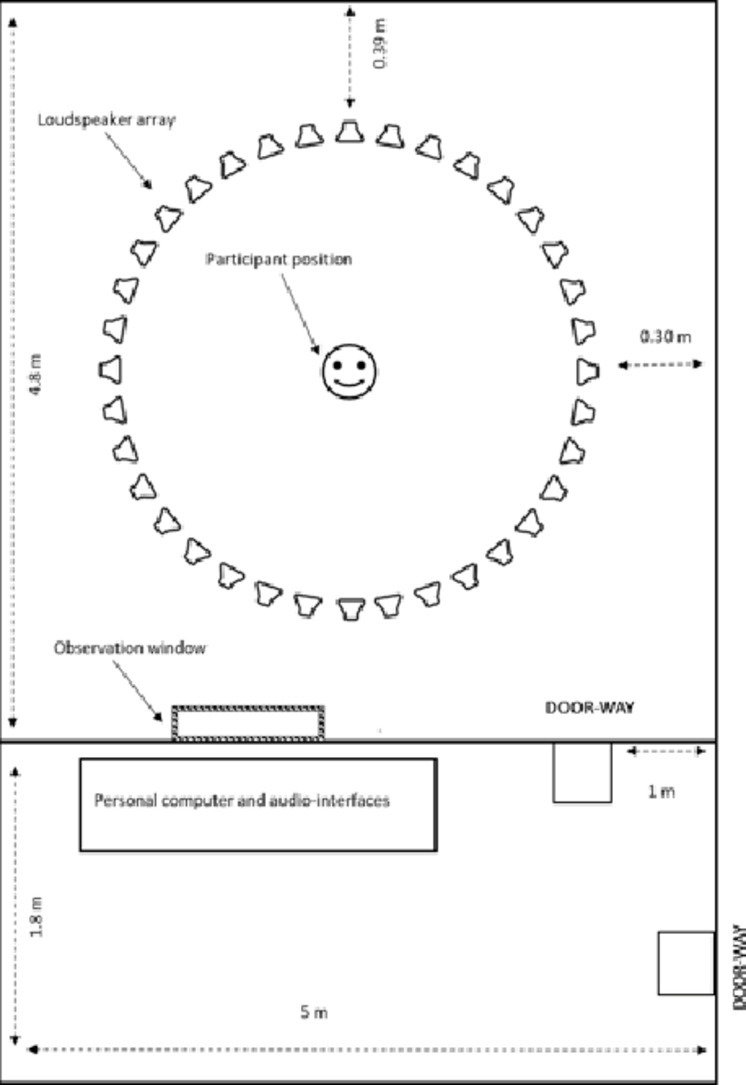

Specifications table

**Table:** 

Subject Area	Medicine and Dentistry
More specific subject area:	Audiology
Method name:	A system for spatial hearing research
Name and reference of the original method:	N.A
Resource availability:	N.A

## Introduction

Spatial hearing is the ability of the human auditory system to utilize the information concerning the location of the sound source and the sound arrival path for the interpretation of the sound. Auditory localization, an underpinning mechanism of spatial hearing not only assists in orienting to the sound source but also in perceptually segregating the target sound and the interfering noise arriving from different directions [1,2]. The spatial hearing mechanism can improve the speech recognition in noise threshold by >12 dB [3,4] and is compromised in individuals with hearing impairment, older adults, and a subset of children with a central auditory processing disorder [4,5]. Since spatial hearing plays a vital role in successful communication in everyday listening situations, assessment and rehabilitation of spatial hearing become imperative in the treatment of auditory disorders. Thereby stimulating a plethora of research in the field of spatial hearing.

Though spatial hearing is a free field phenomenon, the revolution in binaural audio synthesis techniques has enabled researchers to assess spatial hearing abilities under headphones [6]. The headphone-based studies reproduce spatial information by applying Head-Related Transfer Function (HRTF) filters [7], [8], [9], [10], [11]. Nevertheless, they do not account for individual variations. Furthermore, assessment of auditory localization, a foundation mechanism of spatial hearing would be difficult under headphones, especially for hearing aid users. Therefore, a free-field system is ideal for the assessment of spatial hearing. The free-field spatial hearing system includes experimental control software, a multichannel audio interface, and loudspeakers. However, such a complete system is not readily available in the market. Therefore, the system needs to be customized by considering the research objectives, room dimensions, and the scope for future expansion. One such system is set up at the Department of Audiology & Speech-Language Pathology, Kasturba Medical College Mangalore, Manipal Academy of Higher Education, India. The current setup is designed to assess the auditory localization and spatial hearing acuity with 10° precision in the horizontal plane. This paper provides a brief overview of the setup at our laboratory.

## Overview of the lab

The current lab is housed in a rectangular spaced room about 6.7 × 5 × 2.3 m (length x width x height). The floors and ceiling are constructed using concrete, while bricks are used to construct the walls. The adjacent blocks on either side of the lab have classrooms and no heavy noise generators or sound sources are present around the lab. To avoid background noise, the water and drainpipes were not allowed to pass through the lab.

**Fig. 1:**
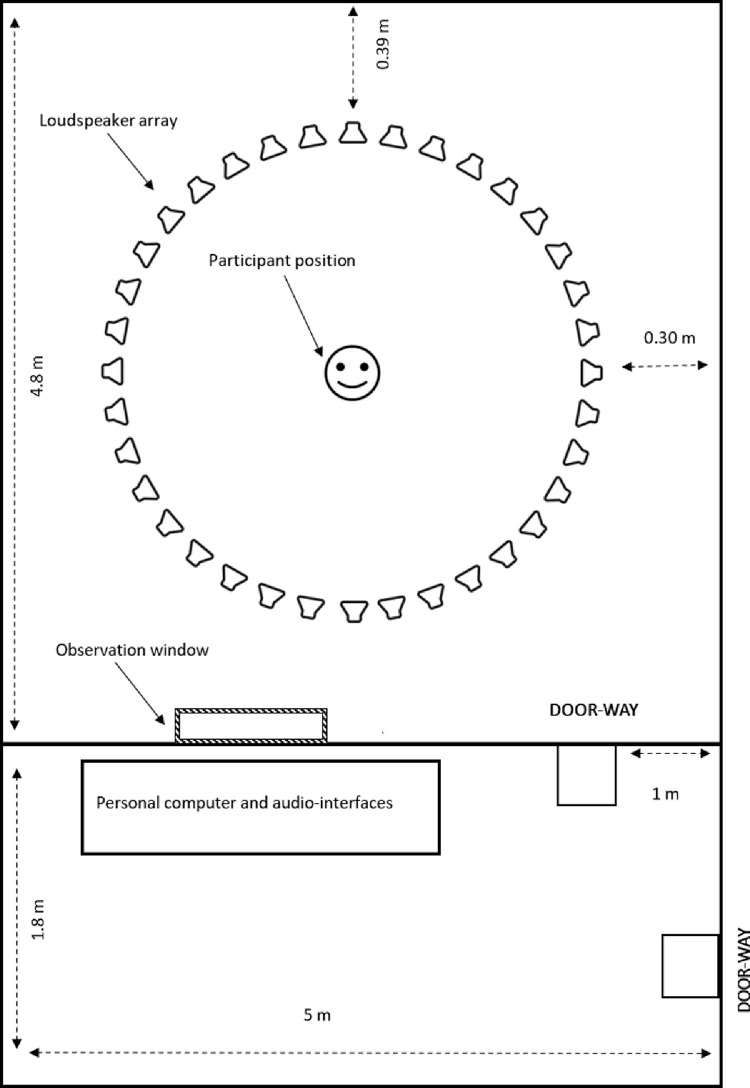
The floor plan of the lab represents the loudspeaker array, subject placement, personal computer, and audio interfaces.

To avoid participant distraction during the experiments, the lab has been divided into an experiment room (4.8 × 5 × 2.3 m) and a control room (1.8 × 5 × 2.3 m). The partition between the experimental and control room has a glass window for direct visual monitoring and an acoustically treated door. The personal computer and soundcards are installed in the control room to reduce the background noise. Instead of personal cable bays, we used universal cable ducts to connect loudspeakers and soundcards, which enable us to modify loudspeaker arrangements with minimal effort (Fig. 1).

## Experimental room

### Room acoustics

**Fig. 2:**
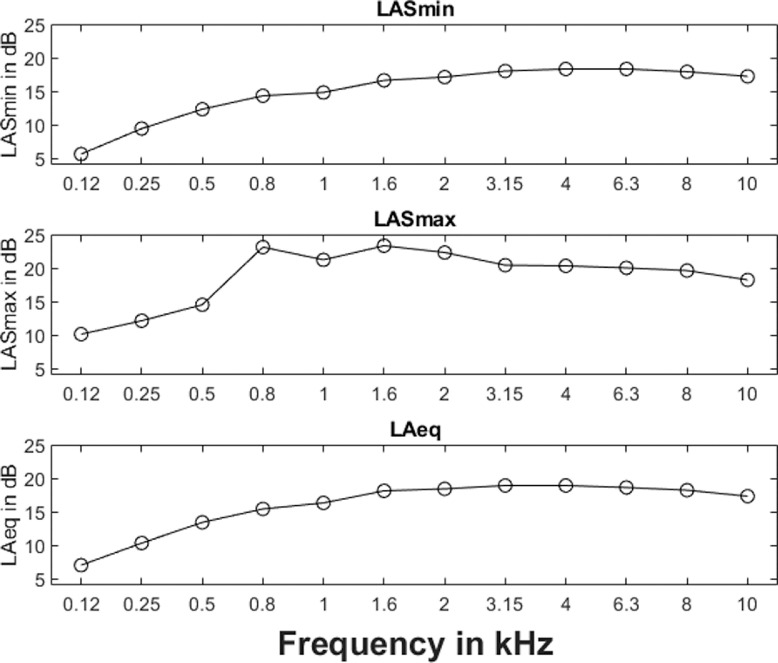
The upper, middle and lower panels depict the minimum, maximum and RMS values of one-third octave band ambient noise levels measured in the experimental room.

The experimental room is acoustically treated. The ambient noise and reverberation time were measured using the M4261 microphone of NTi Audio XL2 sound level meter, positioned at the center of the experimental room. The ambient noise was measured following American National Standard Institute standards [12] and averaged over the timespan of 120 s, yielding a result of 20.8 dBA. The frequency-specific LASmin, LASmax, and LAeq measurements are depicted in Fig. 2.

**Fig. 3:**
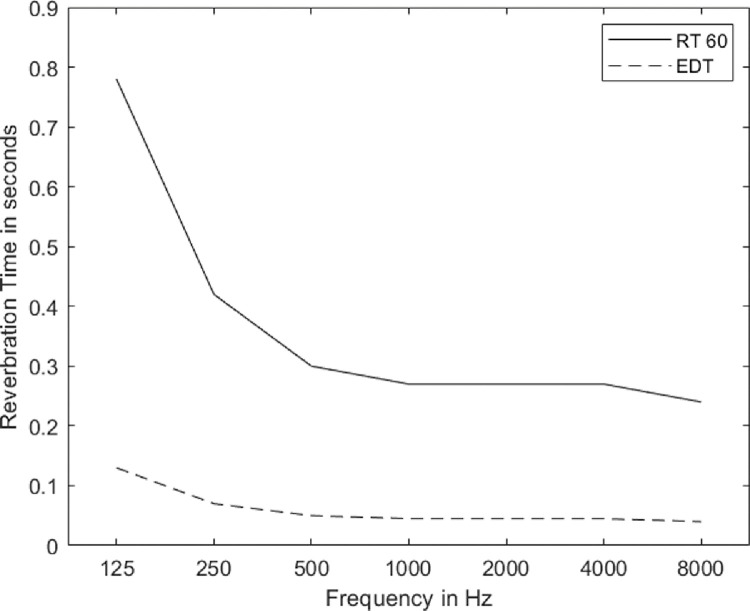
One octave band reverberation time is represented in a solid line and their corresponding early decay time is represented in a dashed line.

The reverberation time (RT60) is defined as the duration required for the intensity to drop by 60 dB after the sound source has stopped [13]. In sound-treated rooms, the recording of complete 60 dB decay is challenging because of ambient noise. To overcome this, T20 has been measured, which is the duration required for the intensity to drop by 20 dB after the sound source has stopped. Once the T20 is measured, the resulting values were multiplied by the factor of 3 to obtain RT60. Further, Early Decay Time (EDT) was calculated by dividing the T20 values by the factor of 2. The findings of the reverberation time measurements for the lab are depicted in Fig. 3.

## Loudspeaker arrangement and seating of participants

**Fig. 4:**
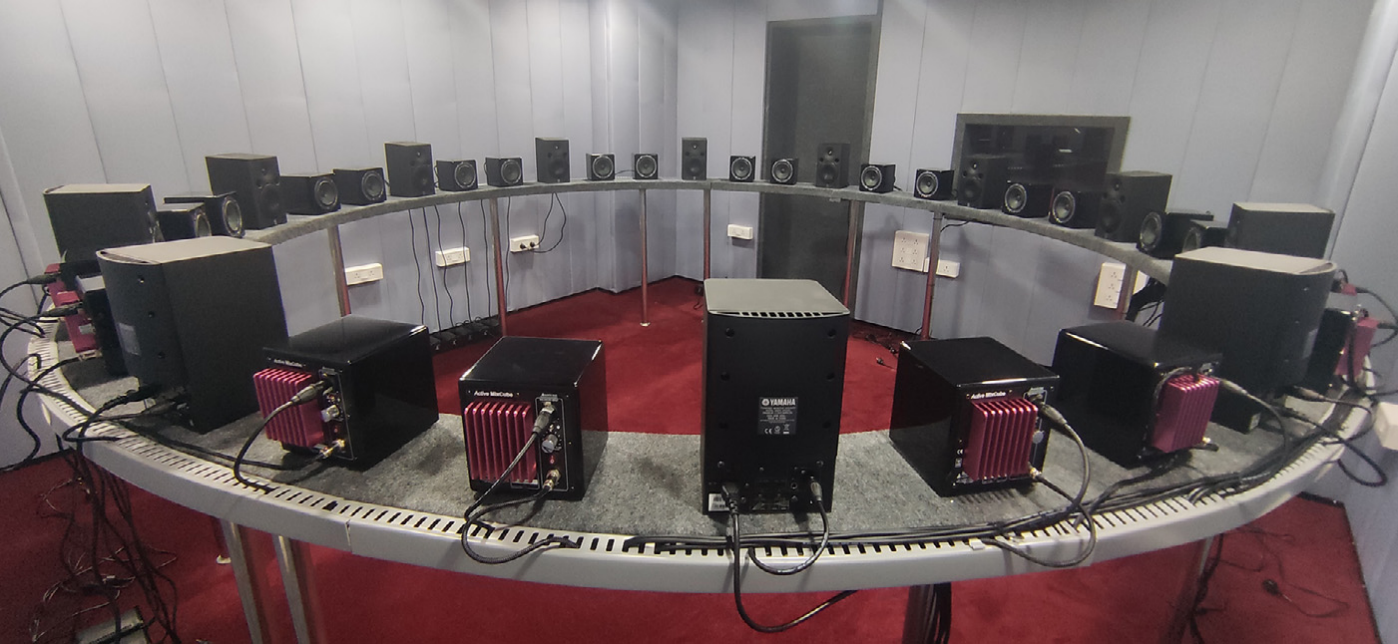
Thirty-six loudspeakers positioned on a circular stand installed in the laboratory.

Thirty-six speakers were positioned circularly on a custom-made plywood ring frame with a width of 8.6 inches and a thickness of 2 inches. The circular frame has a diameter of 3.75 m and a circumference of 11.78 m. It was covered with a layer of an acoustic damper to reduce vibrations and floor-mounted with 12 cylindrical steel rods of identical height, thereby giving the effective height of 1 m to the ring frame. Fig. 4 depicts a pictorial representation of a circular stand with loudspeakers in our lab.

The placement of the participant is determined by considering the far-field hearing phenomenon wherein the ITD and ILD cues remain constant even with an increase in the distance. When the distance between the loudspeaker and the participant reduces below 1 m, the ILD increases, especially, when the loudspeakers are placed in the lateral position. Nevertheless, the ITD cues remain constant irrespective of the distance [14]. Hence, a distance of at least 1 m is required to overcome this unwanted ILD cue. Additionally, a greater distance between the loudspeaker and listener can help in maintaining a stable intensity level, even if the listener makes small head movements [15]. Seeber et al. [15], recommended that the optimum distance to achieve far-field hearing is at least 0.5 times the wavelength of the lowest frequency. However, the maximum possible distance depends on room size. In the current laboratory, the listeners can be placed at a maximum possible distance of 1.87 m from the loudspeakers. This distance would allow the localization judgment experiments involving the signals having a frequency of ≥91 Hz because half the wavelength of 1.87 m corresponds to the frequency of ≈91 Hz (*f* = c/λ where *c* ≈ 344 m/s and *λ* = 2 × 1.87 m). However, this distance may be slightly greater than the critical distance, where the reflected signal may dominate over the direct signal [16]. At the time of this manuscript preparation, the critical distance was not measured but predicted using the following formula recommended by NTi Audio [17]. The predicted critical distance based on the average RT60 is ≈1.40 m. However, the critical distance (CD) would be slightly more for high frequencies having shorter RT60.

$$CD=2\times \sqrt{\frac{V}{C\times RT60}}$$

Placing the participants beyond the critical distance has some advantages as well, especially, when measuring the hearing aid benefit using spatial release from masking. Positioning the participant beyond the critical distance may help to simulate a few real-life situations where the reflected sound is stronger than the direct sound [16,18]. Further due to the flexibility of the loudspeaker stand design, the distance can be adjusted to suit the experimental needs. Additionally, the input signal can also be high pass filtered to eliminate frequencies causing more reverberation.

## Technical characteristics of the equipment

The requirement for equipment with specific characteristics is determined by the experiments done in the lab. In general, for spatial hearing experiments, compact speakers were advised because they can allow us to fit more speakers into a small space and significantly reduce the acoustic reflections produced by the speakers. Often the compact studio monitor speakers are preferred for localization experiments. Most commercially available studio monitor speakers use multiple drivers to produce low and high frequencies separately. The cross-over determines the range of frequencies to be reproduced by each driver. However, at the cross-over point, some frequencies would be reproduced by both drivers leading to unpredictable phase distortions. Also, the multiple drivers in a speaker will act as different points of sources, introducing undesirable localization cues. Phase distortion at the cross-over is not a major factor for the experiments insensitive to phase. Also, the problem of multiple sound sources can be overcome by positioning the speakers far or filtering the input signal. On the other hand, single driver speakers are free of cross-over and multiple source effects. However, the single driver speakers do not have a flat frequency response whereas speakers with multiple drivers produce a relatively flat frequency response.

In the current setup both single and multiple driver speakers are used. Thirty-six active loudspeakers including 24 Avantone Mixcube (Avantone Pro Inc., Tallman, NY) and 12 Yamaha MSP5 (Yamaha music Pvt. Ltd, India) were installed in the current setup. Avantone Mixcube is a single driver speaker whereas the Yamaha MSP5 is a two-way speaker having a woofer and tweeter to reproduce low and high frequencies respectively. The sensitivity of the speakers is adequate to perform unaided listening experiments in individuals having up to a moderately severe degree of hearing loss and aided listening experiments up to a profound degree of hearing loss. The technical characteristics of Avantone and Yamaha studio monitors were represented in Table 1.

**Table 1:** Loudspeaker technical characteristics.

	Avantone Mixcube	Yamaha MSP5
Dimension (h*w*d)	6.5 × 6.5 × 6.5	11×7 × 8.1
Type	Single driver made of paper cone	Dual driver, low frequency driver made of paper cone and high frequency driver made of titanium dome
Frequency response	90 – 17,000 Hz	50 – 40,000 Hz
Maximum output level	104 dB SPL @ 1m	101 dB SPL @ 1m
SNR	113 dB	≥94 dB

Experiments involving two-way speakers will be carefully designed by considering their crossover frequency. The crossover frequency represents the frequency at which the transition occurs between the drivers, which is 2.5 kHz for Yamaha MSP5. So, these speakers will be used to deliver either low or high-pass filtered signals with a cutoff frequency of 2.5 kHz. This allows only one driver of the speaker to be active at any given time, thereby overcoming the phase distortions. The high-frequency driver of Yamaha MSP5 is positioned at a height above the total height of Avantone Mixcube which would act as a source cue. Therefore, these drivers are kept unused when used along with single driver speakers. These modifications in loudspeaker arrangement can be done with minimal effort since our lab uses XLR cables with universal cable ducts for all the speakers.

Typically, the single driver speakers do not have a flat frequency response, and their sensitivity and frequency response might drift over time depending on their usage. As a result, it needs to be equalized across the speakers in terms of both sensitivity and frequency regularly to avoid their potential contribution to measured performance. For this objective, a digital equalization filter was designed using Gaussian white noise (sampling frequency of 44,100 Hz) delivered through loudspeakers and recorded using an omnidirectional microphone (M4261) placed at the subject position.

**Fig. 5:**
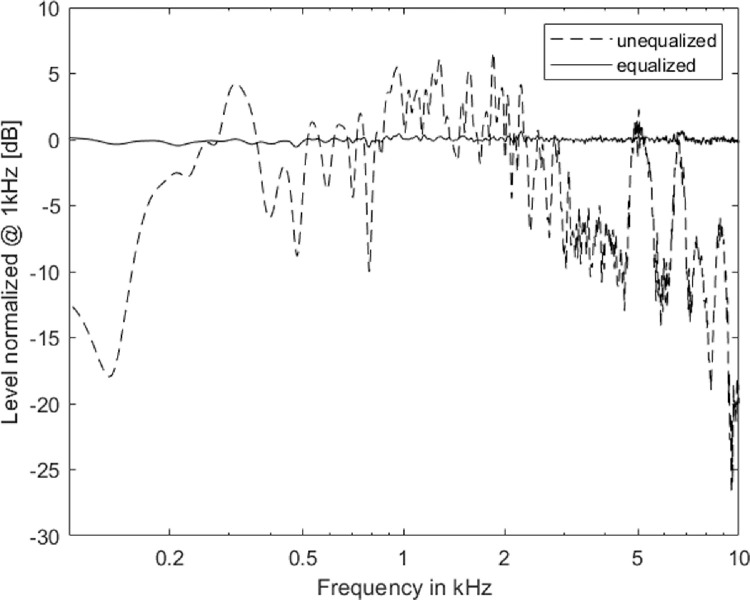
Frequency response of Avantone Mixcube speaker without equalization (dotted line) and after equalization (dashed line). The levels at 1 kHz were normalized to 0 dB.

**Fig. 6:**
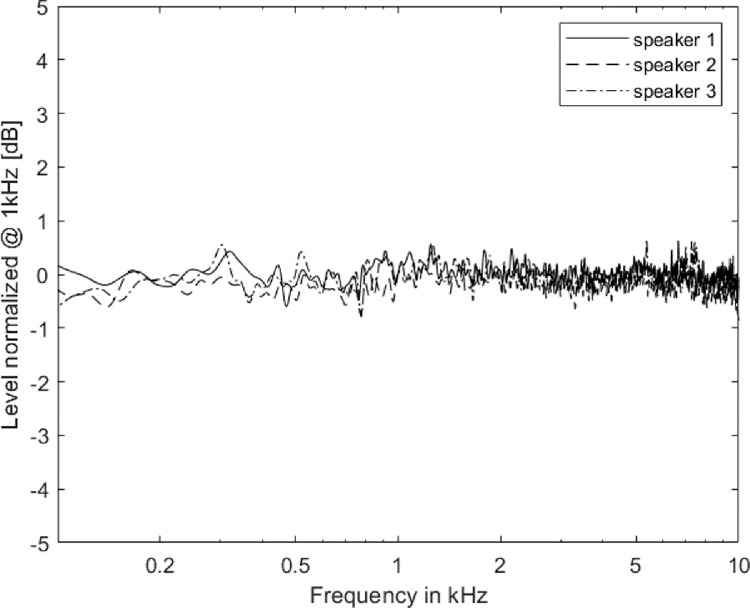
Frequency response of three different speakers after equalization and the levels are normalized at 1 kHz to 0 dB.

The equalization was done in two steps. First, the intensity across the speakers was equalized digitally in MATLAB. Second, the magnitude equalization was performed using a 1024th order Finite Impulse Response (FIR) filter. The filter coefficients were saved for each speaker, which was used to equalize the speaker outputs during experiments. The filtering of signals during equalization attenuated the overall intensity of the signal, hence the RMS amplitude of processed signals was equalized. As a result, we obtained 91.4 dBA and 90.2 dBA for the Avantone Mixcube and Yamaha MSP5 respectively, while maintaining a flat frequency response of ± 2 dB between 100 and 10,000 Hz (please refer to Fig. 5) and within ± 0.6 dB across speakers (please refer to Fig. 6).

The loudspeakers were controlled using two MOTU audio interfaces (MOTU Inc., Cambridge, MA, USA), which are housed in the control room. Both audio interfaces MOTU 24Ao and MOTU 16A can deliver balanced outputs up to 96 kHz sampling rates. Since we use two audio interfaces, they will operate on different digital clock settings resulting in output delays. We have synchronized the digital clock of MOTU 24Ao with MOTU 16A using a Cat-6 Ethernet cable. Alternately, the audio interfaces can be connected to a local network via MOTU AVB switch for precise control over the clocking and latency optimization. MOTU AVB switch streams the audio signals at 1 Gbps with minimal network latency and provides a unified digital clock that allows synchronization in nanosecond precision.

In our lab, we use the Digital Signal Processing toolbox of MATLAB (The Mathworks, Natick, USA) to design spatial hearing experiments on a Lenovo P340 workstation with an Intel Xeon W-1250 (3.3/4.7 GHz clock speed and 12 MB cache) and 64 GB non-ECC RAM. The subject responses can be acquired either by using a manually controlled response application designed using MATLAB application designer or by recording the verbal responses using a wireless microphone. For playback, the ASIO4ALL sound driver is chosen over the default windows driver, since the former is more efficient to handle multi-channel audio signals and enables a connection of the audio interfaces directly to the software thereby reducing the overall output latency. To implement EEG recordings in the future, we installed a computer with such computational specifications. Currently, it is sufficient to generate and deliver multi-channel audio signals as well as apply equalization filters on the fly without any disturbances.

## Applications

The spatial hearing lab setup is currently used to investigate spatial hearing acuity using the RMS localization error metric and spatial release from masking (SRM). The setup enables RMS error and SRM measurements in unaided listening conditions in individuals with a moderately severe degree of hearing loss or less. Nevertheless, aided listening experiments can be performed up to the profound degree of hearing loss.
